# Urinary exosomal miRNA signature of IgA nephropathy: a case–control study

**DOI:** 10.1038/s41598-023-47751-z

**Published:** 2023-12-04

**Authors:** Mythri Shankar, Aditya Shetty, Madhura N.S., Sreedhara C.G., Kishan A., Karthik Tennankore

**Affiliations:** 1 Department of Nephrology, Institute of NephroUrology, Bengaluru, India; 2 Department of Biochemistry, Institute of NephroUrology, Bengaluru, India; 3 University of Dalhousie, Halifax, Canada

**Keywords:** Genetics, Clinical genetics, Genomics

## Abstract

IgA nephropathy is the most common primary glomerulonephritis worldwide and can progress to end-stage kidney disease (ESKD). The current “gold standard” for diagnosis is kidney biopsy, which is invasive and associated with morbidity. miRNAs are small, non-coding endogenous RNA that may serve as non-invasive biomarkers, and that are found in urinary exosomes. Thus far, there is a paucity of studies of the miRNA profile for the diagnosis of IgA nephropathy. Hence, we aimed to study the urinary exosomal miRNA signature of Indian patients with IgA nephropathy. Fifty biopsy-proven IgA nephropathy patients, 50 healthy controls and 25 patients with ESKD (IgA nephropathy) were recruited over 2 years (2020–2022). Urinary exosomes were isolated from which miRNA was extracted . Analysis of urinary exosomal miRNA was done using the digital multiplexed nCounter® human v3 miRNA Expression Assay which contains 799 unique miRNA barcodes. Candidate miRNAs were identified using Lasso regression and consensus clustering. The mean age of IgA nephropathy patients was 36.32 ± 3.067 years, mean creatinine was 2.26 ± 0.318 mg/dl and mean proteinuria was 2.69 ± 0.64 g/day. Compared to healthy controls, the majority (N = 150) of miRNAs were significantly downregulated. Five candidate miRNAs (hsa.miR.146b.3p, hsa.miR.599, hsa.miR.4532, hsa.miR.664b.5p and hsa.miR.221.5p) were able to differentiate between IgA nephropathy cases and controls (AUC > 0.90); the presence of all 5 was associated with 100% specificity and sensitivity for diagnosing IgA nephropathy cases. This study of Indian patients identified that there was a significant difference in the urinary exosomal miRNA profile between IgA nephropathy cases and healthy controls, suggesting that miRNAs may be valuable in the non-invasive diagnosis of IgA nephropathy.

## Introduction

The most common cause of primary glomerulonephritis worldwide is IgA nephropathy^1^. Worldwide, it has an incidence of 2.1 per 100,000 population and commonly affects those in the second or third decades of life^2^.

IgA nephropathy has a progressive clinical course, often characterized by proteinuria, microhematuria and progressive loss of kidney function. Approximately 15–40% reach end-stage kidney disease [ESKD] requiring maintenance hemodialysis by 10–20 years if not diagnosed and treated early^3,4^.

The “gold standard” for diagnosing IgA nephropathy is kidney biopsy, a procedure that is difficult to repeat frequently given its invasive nature and potential morbidity^5^. Complications following kidney biopsy can be as high as 6.4% and include post-procedure bleeding (in approximately 1.2%)^6–8^.

miRNAs are a group of endogenous non-coding RNAs that are 20–25 nucleotides in length. The complementary base pairing of the target mRNA 3″ untranslated region is important to affect the level of gene expression translation, because of which the target mRNA can be inhibited or degraded^9^. They play a crucial part in the development, identification, and management of kidney diseases. miRNA offers benefits as a diagnostic tool due to its stability under challenging conditions, such as boiling, varying pH levels, prolonged storage, and numerous freeze–thaw cycles. Their resistance to endogenous RNAase is attributed to their diminutive size and enclosure within lipid or lipoprotein structures like exosomes^10^.

All these characteristics and the non-invasive nature make miRNAs ideal and potential biomarkers to detect or monitor various human diseases, however there are some limitations. miRNAs in urine sediments are mostly of low quality and easily degrade due to the abundance of RNase in the kidneys, bladder and urinary tract. In contrast, intact miRNAs are abundant in urinary exosomes which are resistant against degradation by RNAse. In contrast to renal biopsy, which only provides a small sample from the kidney, urinary exosomes represent the entire urinary system^11,12^.

Recently, the examination of miRNA levels in urinary sediment has been investigated as non-invasive indicators for kidney diseases, including IgAN^13^. Preliminary studies have indicated that several miRNAs in the urine undergo significant alterations in IgAN. For instance, miR-200a, miR-200b, and miR-429 have been found to be downregulated in IgAN, while miR-29b, miR-29c, and miR-93 levels have been associated with kidney function and the degree of histological damage^14^. Although the available data on urinary miRNA is valuable, it is fragmented and predominantly focused on a few target miRNAs across various ethnic groups; to our understanding, there's no data specific to the Indian population. It is well known that IgA nephropathy is the most common primary glomerulonephritis and has a progressive course in Asian population.

Thus, in a cross-section of Indian patients located in Bengaluru, India, the purpose of this study is to identify the urinary exosomal miRNA profile in patients with IgA nephropathy.

## Materials and methods

### Study design and population

This prospective clinical case–control observational study was conducted in the Department of Nephrology, Institute of Nephro-urology, Bengaluru from September 2020–2022. All methods were carried out in accordance with the Declaration of Helsinki. All experimental protocols were approved by the Institute of Nephro-urology ethical committee (INU/IEC/2020/1). Informed consent was obtained from all participants and/or their legal guardians.

The study population consisted of a total of 125 individuals from whom urine samples were collected from which to extract miRNA. The cases consisted of 50 native biopsy-proven IgA nephropathy (IGAN) patients during the study period, 50 Healthy controls (CTL) consisting of healthy volunteers between 18 and 65 years old with normal renal function and normal urinalysis without a personal or family history of nephropathy or other serious illnesses were recruited and 25 ESKD patients with biopsy-proven IgA nephropathy. The cases were identified prospectively and consecutive kidney biopsies that showed IgA nephropathy were recruited. Fresh urine sample was collected within few days of kidney biopsy report.

Healthy controls were recruited prospectively, and included adults between 18 and 60 years of age. Healthcare workers and patients care takers were screened and included as healthy volunteers.

Patients with a concomitant diagnosis of diabetes, urinary, respiratory, or gastrointestinal tract infection, chronic hepatic disease, systemic lupus erythematosus, and rheumatoid arthritis were excluded. Patients with crescentic IgA nephropathy and IgA vasculitis were also excluded from the study.

Following, written informed consent, a freshly voided urine sample was collected for exosomal miRNA extraction. The demographic and clinical data of all included IgAN patients, such as age, gender, 24-h urinary protein excretion (UPE), and serum creatinine (S.Cr) were recorded at the time of kidney biopsy. The estimated glomerular filtration rate (eGFR) was calculated using the Chronic Kidney Disease Epidemiology Collaboration equations using creatinine 2021^15^.

### RNA extraction and nanostring miRNA expression assay

Exosomal RNA was isolated from human urine samples using Norgen’s Urine Exosome RNA isolation kit (Cat#47200). 2 ml of urine from 5 subjects was pooled to obtain 10 ml of pooled urine samples for the Control group. Similarly, 5 ml of urine from 2 subjects was pooled for IgA Nephropathy (IgAN) cases. In total, 10 control and 25 IgAN case samples were included for the study. Urine samples were completely thawed and incubated for 5 min at 37 °C to dissolve any sediment. The pooling protocol was followed as per manufacturer guidelines. Following that, RNA was eluted in 0.1 ml of elution Buffer. Eluted RNA was concentrated using Zymo’s RNA Clean & concentrator-5(Cat#R1015). Exosomal RNA was eluted from Zymo column in 15 µl of Elution buffer provided in the kit. Quantitation was performed using Qubit RNA HS assay (Invitrogen, Cat # Q32855) kit and also qualitatively analyzed on Agilent 2100 bioanalyzer Pico chip.

With NanoString nCounter Human v3 miRNA Expression Assay (NS_H_miR_v3b) kit (CSO-MIR3-12), miRNA (3ul) was ligated to mir-Tag with ligation buffer and Ligase supplied with the kit. Ligated product was diluted with 15 µl of nuclease free water and denatured at 85ºC for 5 min. 5 µl of this was hybridized overnight at 65 °C with Reporter and Capture probes. Protocol followed as per Manual (nCounter miRNA Expression Assay User Manual, MAN-C0009-07)^16^.

Post hybridization, samples were analyzed on nanoString nCounter SPRINT machine^17–19^.

### miRNA expression analysis

Every sample was evaluated using the nCounter Analysis System (from NanoString Technologies) and the nCounter Human v3 miRNA Expression Assay (NS_H_miR_v3b) panel, which encompasses 799 unique miRNA barcodes for endogenous miRNA^20^. The housekeeping genes incorporated in the panel include beta-actin (ACTB), beta-2-microglobulin (B2M), glyceraldehyde 3-phosphate dehydrogenase (GAPDH), ribosomal protein L19 (RPL19), and Ribosomal protein lateral stalk subunit P0 (RPLP0). Additionally, the panel contains SpikeIn miRNAs, such as Arabidopsis thaliana miR159a (ath-miR159a), Caenorhabditis elegans (cel)-miR-248 and miR254, and Oryza sativa (osa)-miR414 and osa-miR442, along with positive and negative controls to evaluate the overall assay efficiency and monitor the ligation efficiency. The initial miRNA data in RCC (Reporter Code Count) format underwent further analysis using the nSolver analysis software (from NanoString Technologies), version 4.0. QC metrics concerning Imaging, Binding density, positive control, limit of detection, and ligation were verified before advancing to subsequent analyses.Normalization of the raw data was performed using the geometric mean of positive controls and top 100 highly expressed miRNAs. The differential expression between the groups were calculated using the build ratio utility present within the nSolver (foldchange). The thresholds considered for a significant differentially expressed miRNA were, foldchange ≥ 1.2 or ≤ − 1.2, P-value ≤ 0.05 and either of the two groups (test/control) should have their geometric mean expression ≥ average count for negative control probes in the panel.

The ClustVis tool (http://biit.cs.ut.ee/clustvis/) was used for principal component analysis (PCA) and Heatmap generation, using the normalized expression data from the samples used in the study^21^.

The functional enrichment analysis was performed using miEAA 2.0 (https://ccb-compute2.cs.uni-saarland.de/mieaa2/) online server, where over-representation analysis was performed using the list of significant differentially regulated miRNAs as input^22^. Upregulated and downregulated lists of miRNAs were separately analyzed. The options set during the analysis were, P-value adjustment method set to FDR (Benjamini-Hochberg) adjustment, minimum required hits per sub-category set to 2 and significance level set to 0.05. The databases queried against for performing the over-representation analysis include REACTOME (mirPathdb), KEGG, MNDR database for pathway and disease association enrichment while for Gene Ontology Biological Process (BP) and Molecular Function (MF) enrichment, mirPathdb was used.

The Pearson correlation analysis of the normalized expression of miRNAs against clinical variables like Age, eGFR, Serum Creatinine, Blood Urea, 24-h urine protein (proteinuria), Mesangial expansion, Endocapillary proliferation, Sclerosis and IFTA was performed.

### miRNA feature selection using Lasso regression

From the statistically significant miRNAs found during the comparison of IgAN cases against HealthyControlsby nSolver was further used for selection of optimal miRNA using Least absolute shrinkage and selection operator (LASSO) regression^23^.

A minimum lambda value of 0.0057 was used for calculating the coefficients of all the features. Further non-zero features were categorized as Lasso-selected miRNA features. The above steps were completed with the ‘glmnet’ R package^24^.

#### Diagnostic value analysis for Lasso selected miRNA features

Receiver operating characteristic (ROC) curve analysis was performed to evaluate the diagnostic value of selected features by Lasso regression. The current study was used as the discovery cohort for the ROC curve analysis while the GEO dataset GSE64306 was used as the validation cohort^21^. For the ROC curve analysis a classic logistic regression (LR) model along with Leave-one-out cross-validation (LOOCV) was utilized to classify the subject as either IgAN or Control. LOOCV was employed through the caret R package^25^. The AUC values were calculated and visualized using the pROC R package^26^.

#### Consensus clustering of samples using Lasso selected miRNA features

To evaluate the efficiency of selected miRNA features on clustering samples to IgAN and Controls, Consensus clustering was applied using R package ConsensusClusterPlus v.1.62.0^27^. The Euclidean distance was used to calculate the similarity distance between samples, with a hierarchical clustering algorithm used for clustering. The parameters pItem, pFeature and innerLinkage were set to 0.80, 1 and 'ward.D2' respectively with the scheme being executed for 500 times. The best k value of 2 (optimal number of clusters) was chosen on the basis of cumulative distribution function (CDF)^28^.The clusters identified within the samples were visualized using the R software package pheatmap v.1.0.12.

#### miRNA-mRNA-pathway network

For selected miRNA features with high AUC values, experimentally validated mRNA target prediction using miRTarBase v.9.0 database was performed^29,30^.

Further the pathway and disease enrichment analysis for the targets of each miRNA was performed using DAVID web server (https://david.ncifcrf.gov/)^31^. The network of miRNAs, their target genes, related pathways and disease association was constructed using Cytoscape software (https://www.cytoscape.org/)^32^.

### Statistical analysis

All statistical analysis was performed by using R (version 4.0.2, https://www.r-project.org/). The correlation with clinical parameters were explored by the Pearson’s rank correlation coefficient. Receiver operating characteristic (ROC) curves were constructed by standard methods. P value below 0.05 was considered statistically significant.

## Results

The mean age of IgA nephropathy cases was 36.32 ± 3.067 years and 76% were males and 24% were of female sex. Mean creatinine was 2.26 ± 0.318 mg/dl, mean proteinuria was 2.69 ± 0.64 and mean eGFR was 45.46 ± 8.492 ml/min/1.73m^2^ in the IgA nephropathy group (Table 1).

**Table 1:** Baseline characteristics of IgA nephropathy cases, healthy controls and end stage kidney disease patients.

Characteristics	IgA nephropathy (n = 50)	Healthy controls (n = 50)	IgA nephropathy end stage kidney disease (n = 25)
Mean age (in years)	36.32 ± 3.067	39.81 ± 4.13	33.3529 ± 4.228
Male:female	3:1	3:1	2.6:1
Mean S. creatinine (mg/dl)	2.26 ± 0.318	0.8 ± 0.24	10.2847 ± 1.663
Mean eGFR (ml/mib/1.73m2)	45.46 ± 8.492	133.5 ± 53.5	7 ± 1.664
Mean proteinuria (g/day)	2.69 ± 0.64		
M1	98%		
E1	46%		
S1	26%		
T1	38%		
T2	12%		

**Figure 1:**
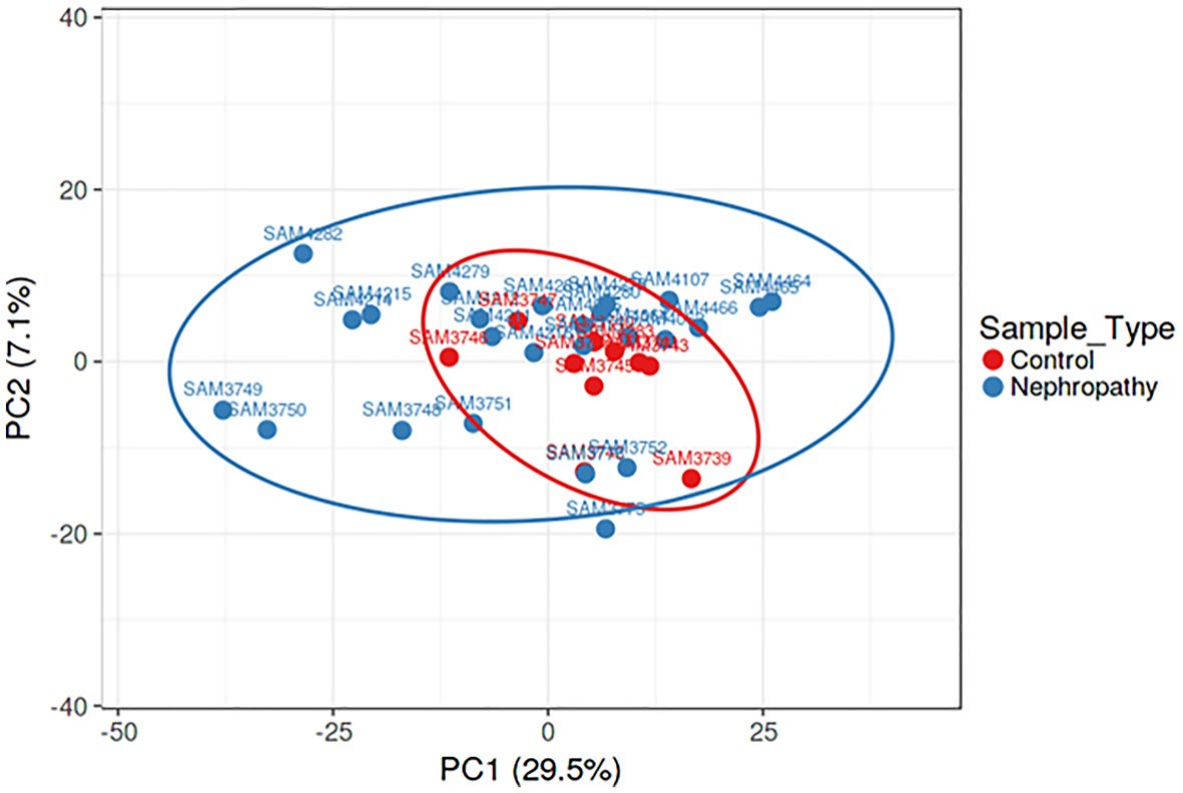
Principal component analysis dimension 1 and dimension 2 of IgA nephropathy samples (blue dots) and healthy controls (red dots).

Principal component analysis (PCA) of miRNA profiles showed that IgA nephropathy samples and samples from healthy control individuals were separate from each other (Fig. 1).

**Figure 2:**
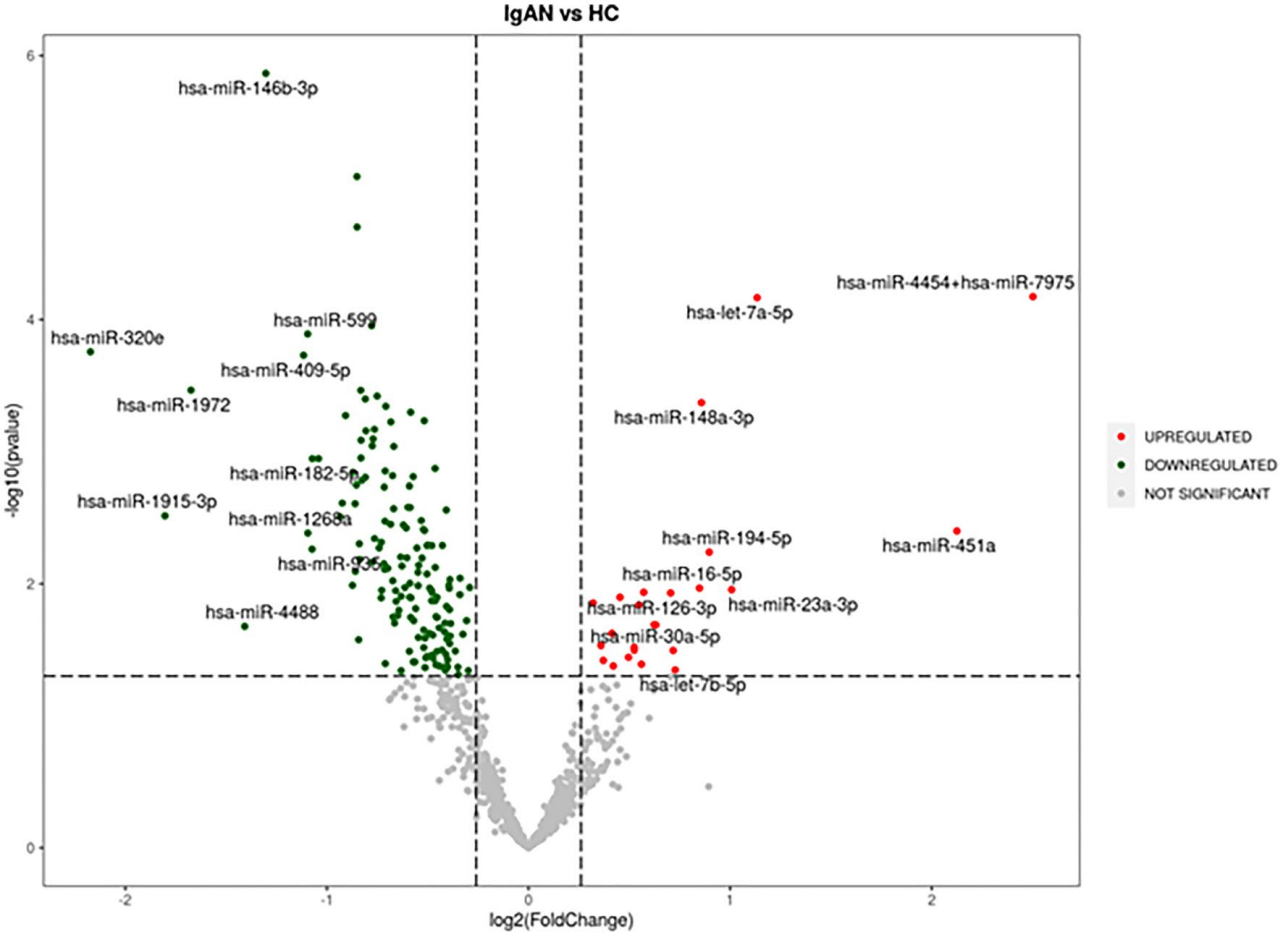
Volcano plot: Significantly upregulated miRNA in IgA nephropathy samples compared to healthy controls are marked in red and downregulated miRNA are marked in green.

174 urinary exosomal miRNAs showed significant differential expression in IgA nephropathy cases compared to the healthy control population. Out of the 174 miRNAs, the majority (N = 150) were significantly downregulated and 24 were significantly upregulated (Fig. 2).

**Figure 3:**
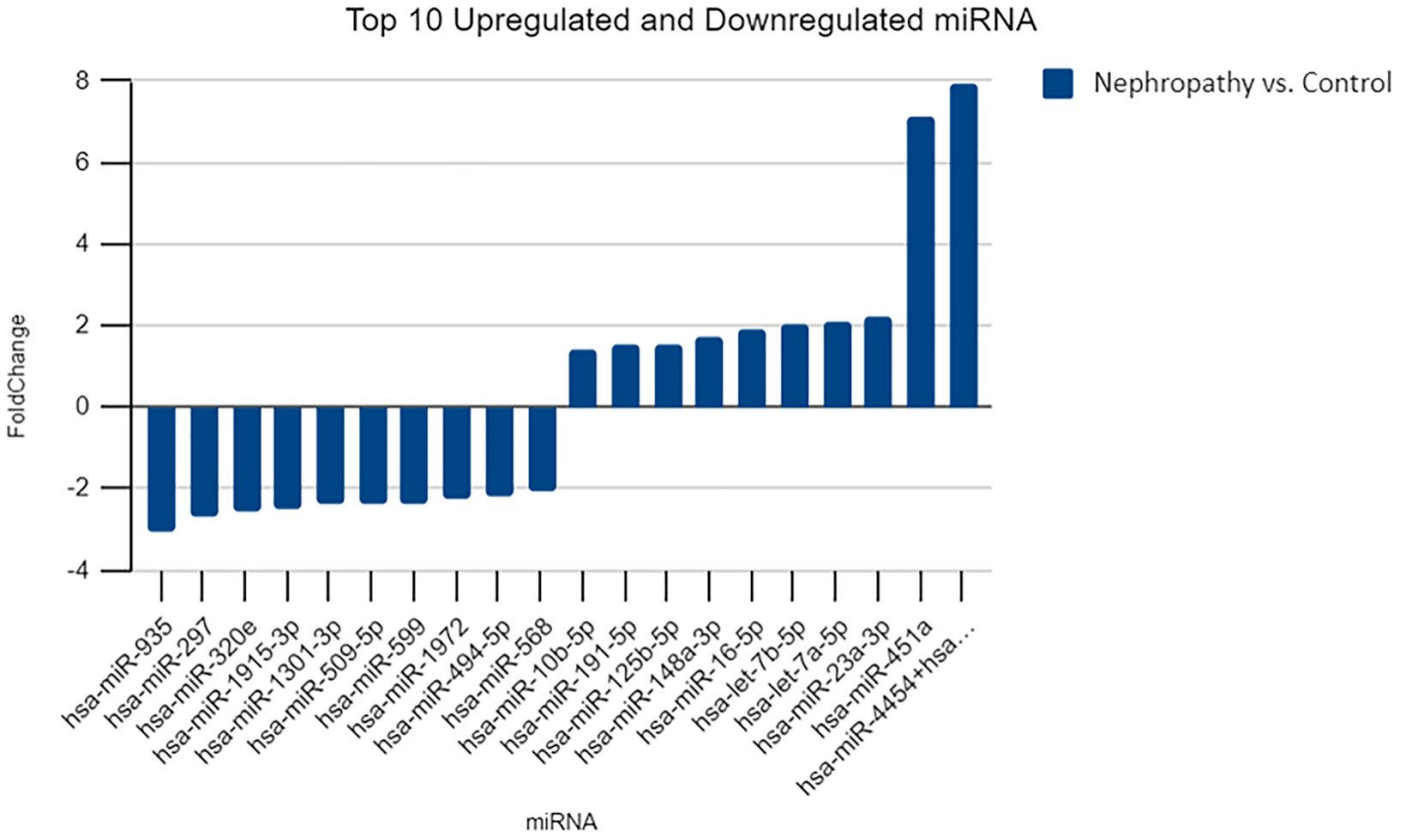
Top 10 upregulated and downregulated miRNA in IgA nephropathy cases compared to healthy controls.

The top 10 significantly upregulated and top 10 significantly down regulated miRNAs are shown in Fig. 3.

**Figure 4:**
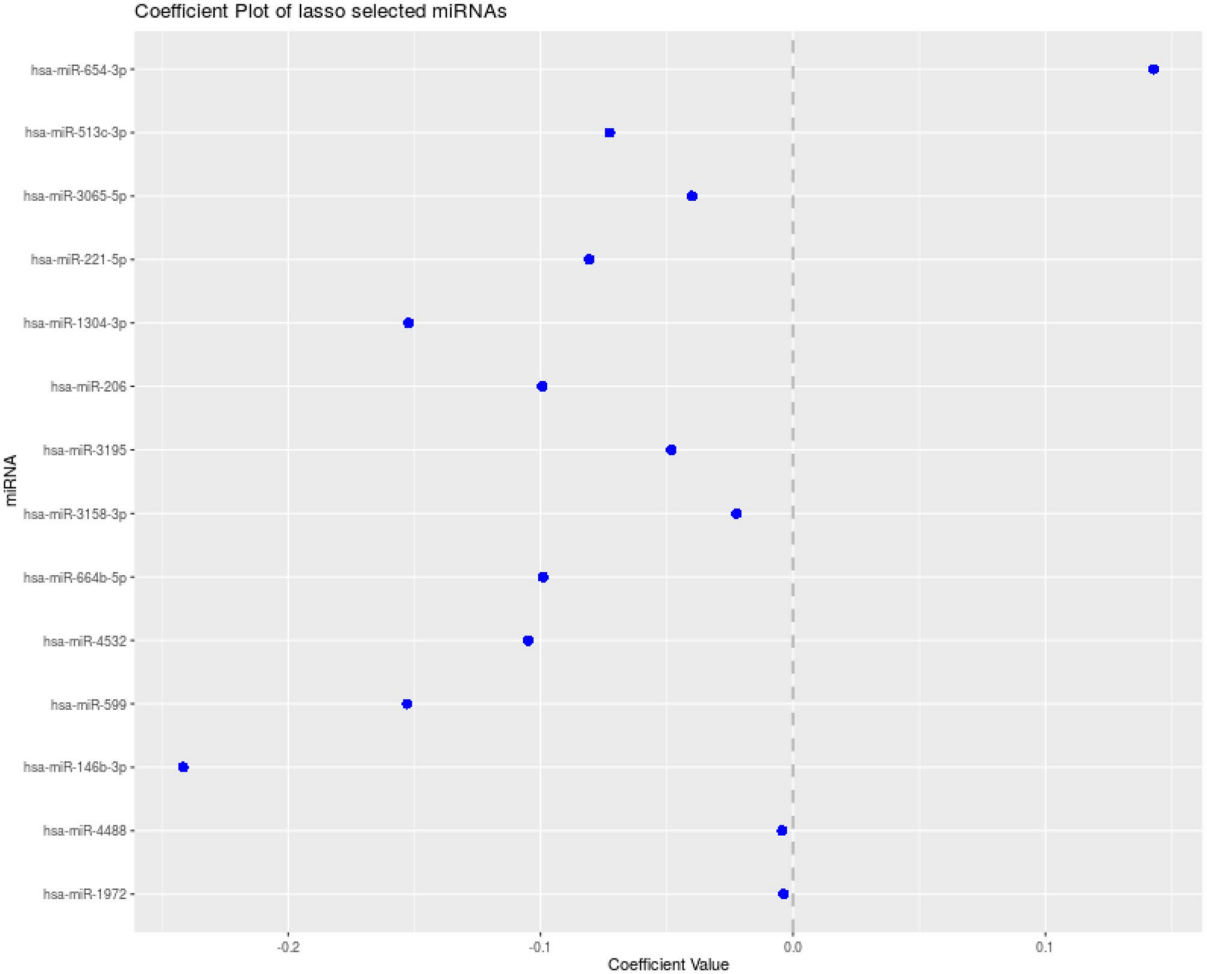
Co-efficient plot of Lasso regression miRNAs.

It was observed after Lasso regression that out of 174, 14 miRNAs were the most important ones (Fig. 4).

**Figure 5:**
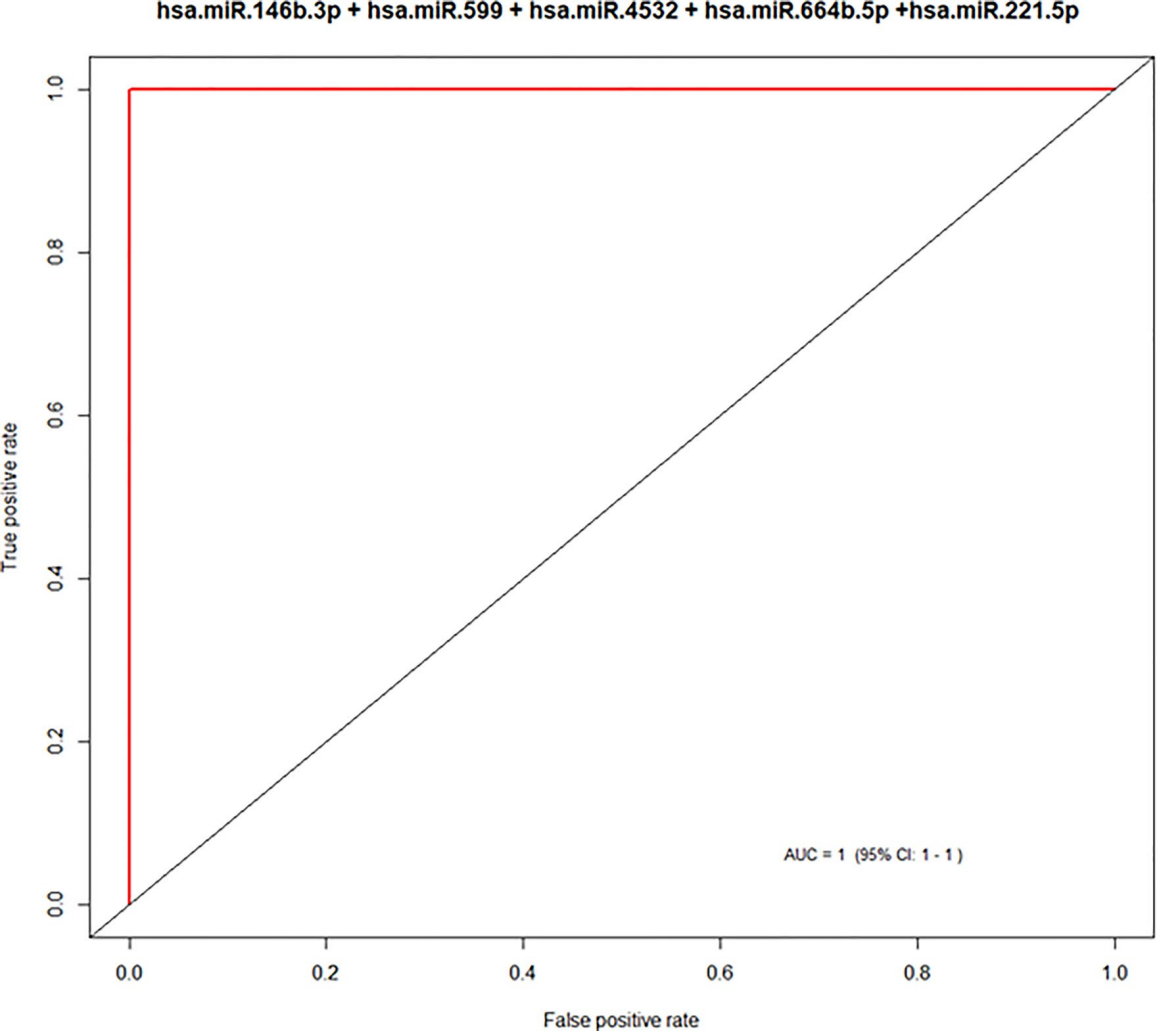
Area under the curve of > 0.9 for the group of 5 candidate miRNAs with a significant potential as biomarkers.

A group of 5 candidate miRNAs out of these 14 validated miRNAs had AUC values > 0.90 giving them a good probability of acting as a biomarker (Fig. 5 and Table 1).

**Figure 6:**
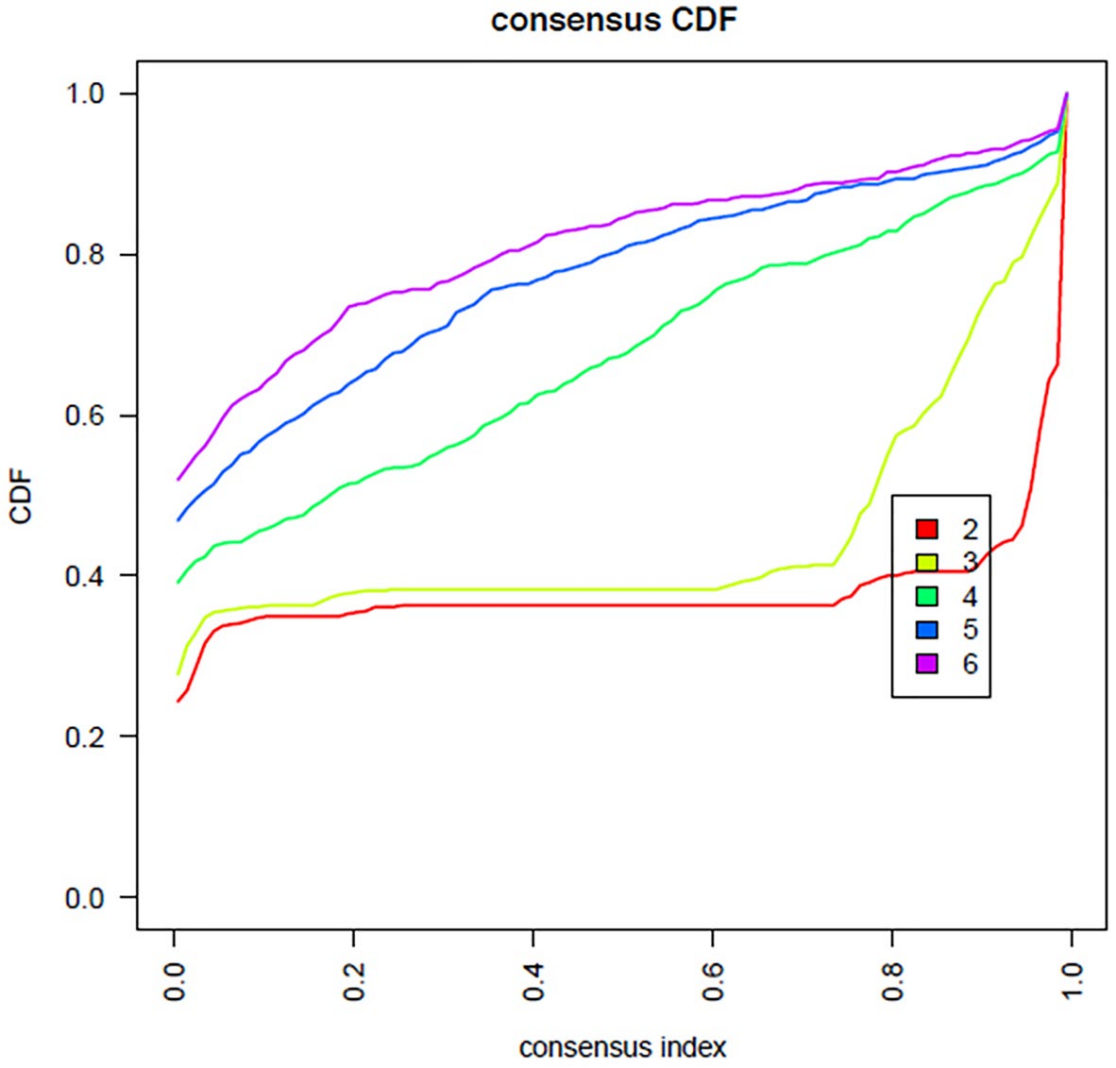
Consensus index—cumulative distribution function shows the red line with two peaks—cluster with two groups using the group of five candidate miRNAs.

**Figure 7:**
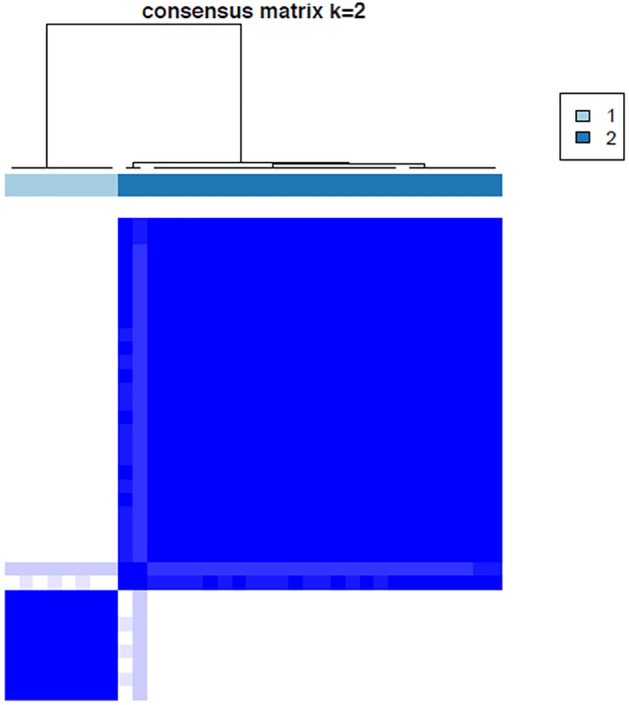
Consensus matrix shows cluster with two groups with the group of five candidate miRNAs.

**Figure 8:**
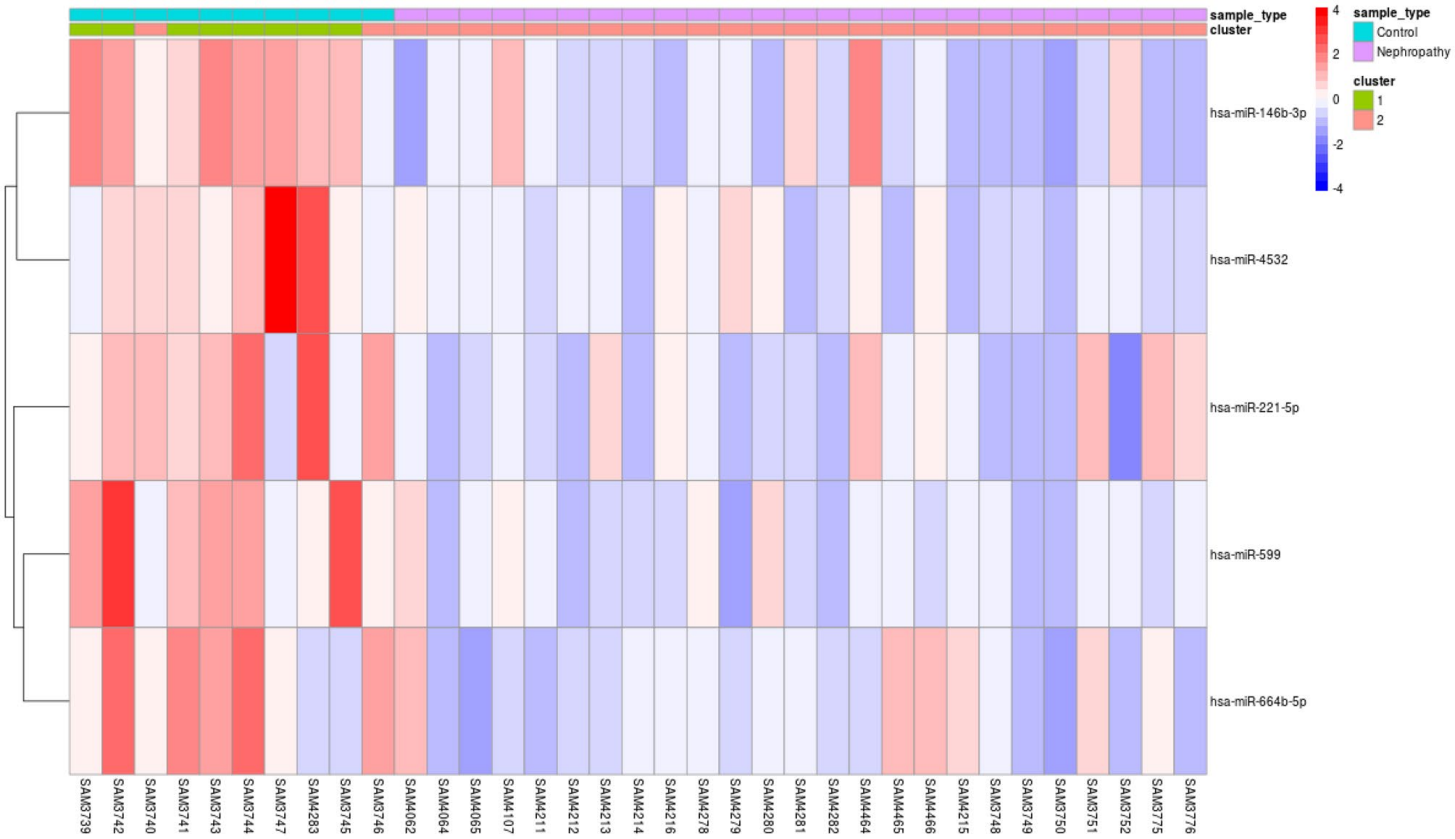
Heat map showing the 5 miRNAs with biomarker potential giving an accuracy of 99.

Consensus clustering was applied to check the efficiency of selected five candidate miRNA on clustering samples to IgAN and Controls. The group of these five important miRNAs which showed high AUC values > 0.90, were clustered into 2 groups. With the exception of two misclassifications among the controls, all remaining samples were correctly assigned to two different groups by the expression of selected candidate five miRNAs together with 99% efficiency. The results have been visualized as Heatmap (Figs. 6,7 and 8).

### Validity of urinary exosomal miRNAs as biomarkers of IgA nephropathy

These five miRNA as a combined group were able to successfully differentiate between healthy controls and Ig A nephropathy cases with excellent sensitivity (100%), specificity (100%), positive predictive value (100%) and negative predictive value (100%) and a diagnostic efficacy of 99%. The area of the receptor-operator curve (AUC) is a measure of discrimination. The closer the value is to 1, higher accuracy of results. When the 5 miRNAs were combined, it showed excellent discrimination from healthy controls with AUC of 1.0 (95% CI 0.7–1.0, Table 2).

**Table 2:** The diagnostic potential of the selected group of five significant miRNAs with biomarker potential.

Model using all 5 miRNAs (hsa.miR.146b.3p + hsa.miR.599 + hsa.miR.4532 + hsa.miR.664b.5p + hsa.miR.221.5p) as feature	
Accuracy (95% CI)	1 (0.7151, 1)
Sensitivity	1
Specificity	1
PosPredValue (PPV)	1
NegPredValue (NPV)	1
Prevalence	0.1818
DetectionRate	0.1818
DetectionPrevalence	0.1818
AUC	1
No. of misclassified samples	0
Wrongly classified samples	NA
Kappa	1

Significant correlation of urinary exosomal miRNA with baseline parameters of IgA nephropathy cases are depicted in Table 3.

**Table 3:** Significant correlation of urinary exosomal miRNA with baseline parameters of IgA nephropathy cases.

Clinical parameter	miRNA	Pearson correlation	P value	Adjusted P value for age
Age	hsa-miR-491-3p	0.772496182	6.04609E-06	0.043423004
egfr	hsa-miR-375	− 0.54406	0.004933	
	hsa-miR-31-5p	− 0.51072	0.009087	
	hsa-miR-664b-5p	− 0.49217	0.01245	
	hsa-miR-141-3p	− 0.46921	0.017971	
	hsa-miR-200c-3p	− 0.43995	0.027755	
	hsa-miR-379-5p	0.412962	0.040198	
	hsa-miR-378 h	0.4311	0.031435	
	hsa-miR-548n	0.435125	0.029715	
	hsa-miR-503-3p	0.457449	0.021494	
	hsa-miR-769-3p	0.476456	0.016047	
	hsa-miR-1288-3p	0.490151	0.01287	
	hsa-miR-320e	0.495641	0.011752	
	hsa-miR-548z + hsa-miR-548 h-3p	0.498938	0.01112	
	hsa-miR-877-5p	0.502804	0.010415	
	hsa-miR-888-5p	0.514417	0.008516	
S. creatinine	hsa-miR-548q	− 0.48525	0.013942	
	hsa-miR-378 h	− 0.46509	0.019149	
	hsa-miR-1306-3p	− 0.44899	0.024358	
	hsa-miR-1288-3p	− 0.43612	0.029302	
	hsa-miR-5010-5p	− 0.43251	0.030824	
	hsa-miR-3918	− 0.41029	0.041637	
	hsa-miR-641	− 0.40747	0.043204	
	hsa-miR-200b-3p	0.424832	0.034271	
	hsa-miR-612	0.460265	0.020604	
	hsa-miR-375	0.527563	0.006724	
24 h urine protein	hsa-miR-22-3p	− 0.49976	0.010966	
	hsa-let-7 g-5p	− 0.45284	0.02302	
	hsa-miR-664b-5p	− 0.44754	0.024878	
	hsa-miR-362-3p	− 0.44636	0.025308	
	hsa-miR-10b-5p	− 0.4404	0.027577	
	hsa-miR-100-5p	− 0.41117	0.041161	
	hsa-miR-31-5p	− 0.40368	0.045375	
	hsa-miR-23a-3p	− 0.40259	0.046015	
	hsa-miR-222-3p	− 0.40199	0.046375	
	hsa-miR-502-5p	0.454568	0.022437	
	hsa-miR-1285-5p	0.458466	0.021169	
	hsa-miR-4284	0.476136	0.016128	
	hsa-miR-206	0.480491	0.015051	
	hsa-miR-888-5p	0.492791	0.012322	
	hsa-miR-548ar-5p	0.506437	0.009787	
	hsa-miR-2117	0.516496	0.008209	
	hsa-miR-1972	0.518825	0.007876	
	hsa-miR-664b-3p	0.633966	0.000667	
	hsa-miR-1286	0.647801	0.000463	
Mesangial expansion	hsa-miR-1276	− 0.554350538	0.004033	
	hsa-miR-148b-3p	− 0.54655137	0.0047	
	hsa-miR-4286	− 0.50720338	0.009658	
	hsa-miR-3161	− 0.474829522	0.016464	
	hsa-miR-549a	− 0.467379209	0.018487	
	hsa-miR-2116-5p	− 0.415819383	0.038703	
	hsa-miR-617	0.50912478	0.009342	
	hsa-miR-370-3p	0.513116038	0.008713	
	hsa-miR-939-5p	0.520636112	0.007624	
	hsa-miR-95-3p	0.533631587	0.00601	
	hsa-miR-145-5p	0.558133594	0.003739	
	hsa-miR-552-3p	0.564853874	0.003262	
	hsa-miR-1268a	0.567029713	0.003119	
	hsa-miR-376b-3p	0.601323816	0.001476	
	hsa-miR-345-5p	0.640731043	0.000559	
	hsa-miR-936	0.660839044	0.000323	
Endocapillary proliferation	hsa-miR-141-3p	− 0.585538186	0.002104	
	hsa-miR-128–1-5p	− 0.552425255	0.00419	
	hsa-miR-765	− 0.55039623	0.004361	
	hsa-miR-378e	− 0.520373969	0.00766	
	hsa-miR-21-5p	− 0.515984397	0.008284	
	hsa-miR-15a-5p	− 0.515499378	0.008355	
	hsa-miR-204-5p	− 0.504783483	0.010069	
	hsa-miR-1246	− 0.501959962	0.010566	
	hsa-miR-548q	− 0.497589026	0.011375	
	hsa-miR-99a-5p	− 0.480267763	0.015105	
	hsa-miR-450b-5p	0.59814067	0.001588	
	hsa-miR-597-5p	0.601822556	0.001459	
	hsa-miR-450a-1-3p	0.615619987	0.001053	
	hsa-miR-384	0.623321915	0.000873	
	hsa-miR-523-3p	0.633910168	0.000668	
	hsa-miR-502-5p	0.652513084	0.000408	
	hsa-miR-1247-5p	0.654903136	0.000382	
	hsa-miR-133a-5p	0.661072747	0.000321	
	hsa-miR-382-5p	0.687505183	0.000146	
	hsa-miR-548d-3p	0.697247218	0.000107	
Glomerulosclerosis	hsa-miR-3918	− 0.440159559	0.02767	
	hsa-miR-1178-3p	− 0.428758656	0.032471	
	hsa-miR-25-3p	0.397451159	0.049134	
	hsa-miR-93-5p	0.432557742	0.030803	
	hsa-miR-601	0.47677605	0.015966	
	hsa-miR-4488	0.488926714	0.013131	
	hsa-miR-423-3p	0.50991998	0.009214	
Interstitial fibrosis and tubular atrophy	hsa-miR-1258	− 0.522373537	0.00739	
	hsa-miR-548n	− 0.478327813	0.015578	
	hsa-miR-100-5p	− 0.455529662	0.022119	
	hsa-miR-455-5p	− 0.447259358	0.024981	
	hsa-miR-378e	− 0.382669327	0.059029	
	hsa-miR-627-3p	− 0.381315756	0.060007	
	hsa-miR-1305	− 0.379140547	0.061605	
	hsa-miR-31-5p	− 0.362001741	0.075369	
	hsa-miR-6511a-3p	− 0.357544996	0.079304	
	hsa-miR-520a-5p	− 0.338430646	0.097972	
	hsa-miR-539-5p	0.40118042	0.046856	
	hsa-miR-331-5p	0.402409074	0.046124	
	hsa-miR-548al	0.407435901	0.043221	
	hsa-miR-363-5p	0.422764289	0.03525	
	hsa-miR-6721-5p	0.426606495	0.033448	
	hsa-miR-199a-3p + hsa-miR-199b-3p	0.443111451	0.026525	
	hsa-miR-891b	0.46766435	0.018406	
	hsa-miR-1233-3p	0.488245573	0.013278	
	hsa-miR-33a-5p	0.501372388	0.010672	
	hsa-miR-499a-5p	0.681817469	0.000174	

*IFTA* interstitial fibrosis and tubular atrophy.

**Figure 9:**
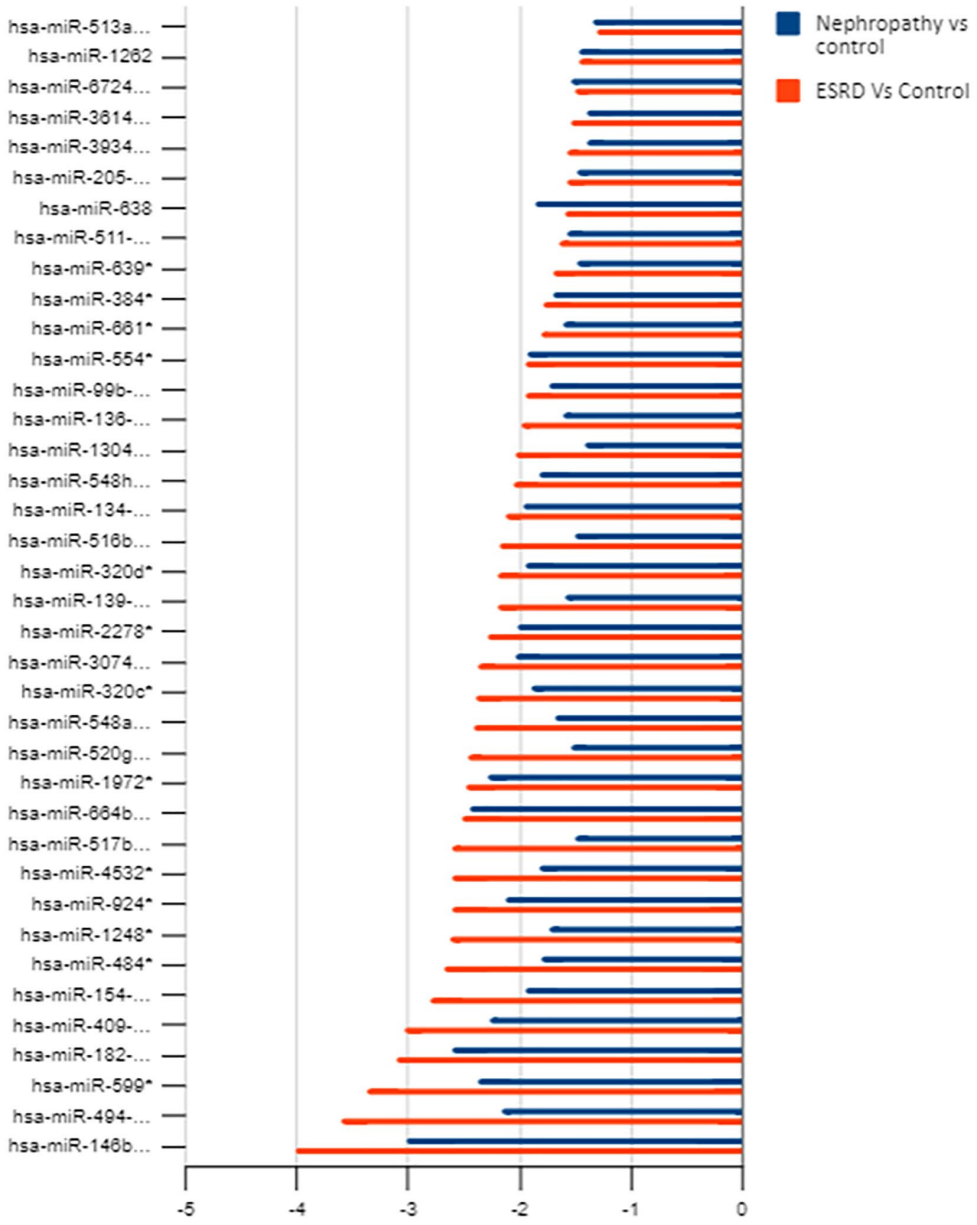
Indicates that the downregulation of miRNAs is increasing in the ESKD condition when compared to IgA Nephropathy.

38 miRNAs were commonly downregulated in both IgA nephropathy cases and ESKD samples, it was found these miRNAs were progressively down regulated more in ESKD samples compared to IgA nephropathy case samples (Fig. 9).

**Figure 10:**
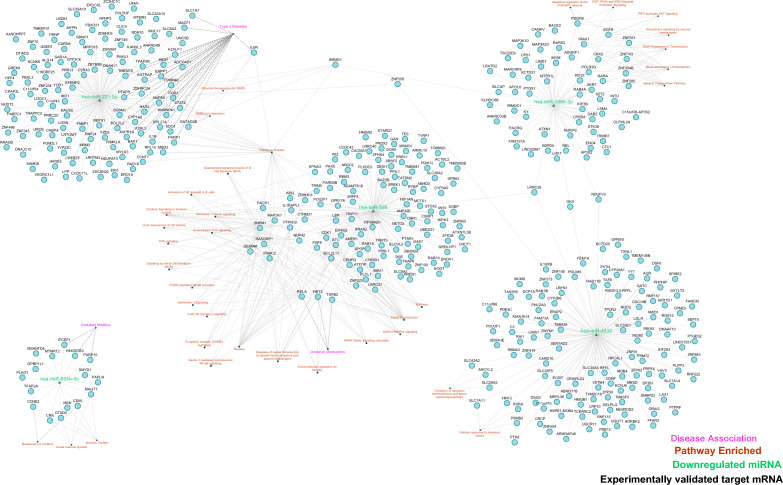
Showing the network of five downregulated miRNA (green), Pathways enriched (in red), experimentally target messenger RNA (black) and disease association (in pink).

In order to gain insight into the biological process regulated by these 5 miRNAs, gene enrichment analysis was performed using DAVID web server (Fig. 10). The results of which are provided in Supplementary Table 1.

## Discussion

Worldwide, IgA nephropathy is the most common primary glomerulonephritis^1^. Unfortunately, there are no biomarkers for the diagnosis of IgA nephropathy. Urinary exosomes are a stable source of miRNAs, which makes urine a potential source for the discovery of biomarkers for kidney diseases. In this study, we discovered a number of differentially expressed urinary exosomal miRNA in patients with IgA nephropathy compared to healthy control population by nanostring technology. We identified that a group of five candidate miRNAs can be used to differentiate between healthy controls and IgA nephropathy.

A handful of genome-wide association studies have been performed on kidney biopsy tissues^33^, peripheral blood mononuclear cells^34^, and urine sediments^35,36^, identifying differential expression of miRNA in IgA nephropathy cases compared to healthy controls. This study is unique as urinary exosomal miRNAs were studied.

174 miRNA showed significant differential expression in this study compared to the healthy control population. Out of the 174 miRNAs, the majority (150) were significantly downregulated, and 24 were significantly upregulated. These results are consistent with the study by Tan et al.^33^, who studied miRNA in kidney biopsy tissue of IgA nephropathy patients and identified 85 differentially expressed miRNA, with the majority (74) being significantly downregulated and 11 being upregulated.

The biological role of the 5 identified miRNA were identified in the following studies:

**Table:** 

hsa-mir-4532	Dysregulated in diabetes mellitus and diabetic nephropathy	^37^
hsa-mir-146b family	Chronic renal insufficiency models have shown that the expression of miR-146a in the kidney and urine is significantly related to inflammatory cell infiltration and interstitial lesions	^38^
hsa-mir-599	Responsible for platelet related complications in chronic kidney disease	^39^
hsa-mir-221-5p	Promising biomarker for lupus nephritis	^40^
		^41^
		^42^

To the best of our knowledge, this is the first study to identify the urinary exosomal miRNA signature in IgA nephropathy patients using nanostring technology and the first study conducted entirely in an Indian population. Only one other study from China^43^, identified urinary exosomal miRNA in IgAN cases. They identified differential expression of miR-215-5p,miR-378i,miR-365b-3p and miR-135b-5p. The results from this study was consistent and showed a significant differential expression in three of these miRNA. i.e. hs-mir-215, hs-miR-378, and hs-miR135b. However, this study from China^43^ had an even smaller sample size of 18 IgA nephropathy patients and used high throughput sequencing for screening followed by the RT-PCR method to identify a select few limited miRNAs.

Nevertheless, this study has a few limitations. First, other glomerular disease controls such as diabetic nephropathy, membranous nephropathy,minimal change disease and other chronic glomerulonephritis were not included in the study. Second, follow-up studies are required to assess the prognosis of the disease.

## Conclusion

This study identifies urinary exosomal miRNA signature in IgA nephropathy patients for the first time. The urinary exosomal miRNAs **hsa.miR.146b.3p + hsa.miR.599 + hsa.miR.4532 + hsa.miR.664b.5p + hsa.miR.221.5p**. may serve as novel biomarkers of IgA nephropathy. This study provides insights into the pathogenetic mechanisms of IgA nephropathy, which may shed light on future therapeutic options and paves the way for interesting future research on miRNA in IgA nephropathy.

### Supplementary Information

Supplementary Table 1.

## Data Availability

The datasets generated and analysed during the current study are available in the Gene Expression Omnibus repository, [GEO, https://www.ncbi.nlm.nih.gov/geo/ under the accession number GSE244625].
